# The durability, functionality and acceptability of novel screened doors and windows after 4 years of use in a Gambian village: a cross-sectional survey

**DOI:** 10.1186/s12936-022-04087-9

**Published:** 2022-02-23

**Authors:** Fiona C. Shenton, Musa Jawara, Majo Carrasco-Tenezaca, Jakob Knudsen, Umberto D’Alessandro, Steve W. Lindsay

**Affiliations:** 1 Department of Biosciences, Durham University, Durham, UK; 2 Medical Research Council Unit, The Gambia, London School of Hygiene and Tropical Medicine, Fajara, The Gambia; 3 The Royal Danish Academy of Fine Arts, School of Architecture, Design and Conservation, The School of Architecture, Copenhagen, Denmark

**Keywords:** House screening, Durability, Functionality, Acceptability, The Gambia

## Abstract

**Background:**

The World Health Organization recommends house screening as a tool for malaria control, yet evidence of the long-term durability, functionality and acceptability of this intervention is lacking. In this study, the sustainability and use of novel types of screened doors and windows was examined 4 years after installation in a Gambian village.

**Methods:**

A survey of 31 houses, each with two screened doors and two screened windows, was conducted in the rainy season. There were four types of screened door and two types of screened window. Trained staff carried out the survey and interviews of room owners were conducted in the local language before translation into English.

**Results:**

Structurally, the manufactured doors and windows were highly durable and in excellent condition. Most doors shut smoothly 50/61 (82%), although only 25/61 (41%) shut fully automatically with the latch slotting into the hole on the frame and holding fast. Door locks were less robust, with only (24/61) 39% present and working. Blinds proved especially flimsy, with only 4/109 (4%) of door blinds and 10/56 (18%) of window blinds present and in working order. Householders hung curtains inside most doors 50/61 (82%) and in 26/61 (43%) of the windows. Front doors were commonly found propped open 21/31 (68%) and 23/27 (85%) of those with a front door curtain, put their curtains down at night. Doors and windows were well liked, 19/31 (61%) of respondents were happy with them because they kept mosquitoes out 14/31 (45%) and provided security 12/31 (39%). The main reason given for the use of curtains was to provide privacy 26/28 (93% of those with curtains), especially while the door was open or had ‘see-through’ panels.

**Conclusions:**

Overall, the screened doors and windows were in full-working order and undamaged after 4 years of use. The doors and windows were well liked, especially for their ability to reduce the entry of mosquitoes and for the security they afforded. Improvements to the lock design are needed before scale-up. Most householders hung curtains behind their doors for privacy. Installation of screening in buildings should be accompanied with recommendations that at night, when doors and windows are closed, curtains be lifted or drawn to one side—to improve ventilation and keep the house cool.

**Supplementary Information:**

The online version contains supplementary material available at 10.1186/s12936-022-04087-9.

## Background

The World Health Organization (WHO) has recommended that houses should be screened to enhance protection against malaria [1]. This is encouraging, especially since the prevalence of house screening is increasing in some countries. In Tanzania, house screening is increasing in urban areas [2, 3] and in some rural areas a quarter of windows are screened [4]. Indeed, this practice may be wider than reported in sub-Saharan Africa, but it is poorly documented. The durability of house screening has been measured over relatively short time periods of 6–12 months [5, 6] but none have looked at the longer-term durability, functionality and acceptability of this intervention.

**Fig. 1:**
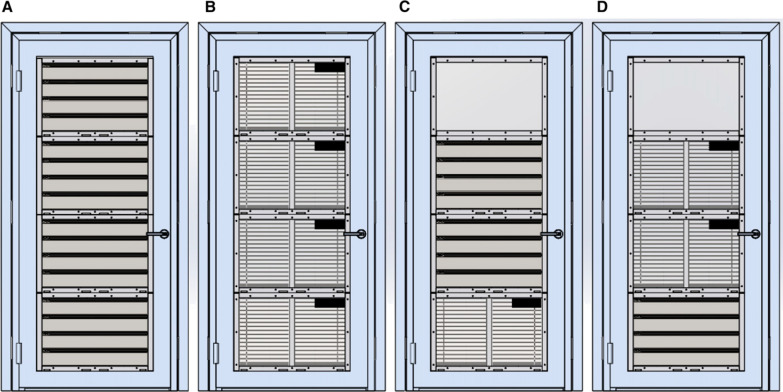
Prototype doors (internal view). Where **A** is the concertinaed door, **B** is the blinds door, **C**, **D** are doors with a combination of panels and translucent windows at the top. Source: Jawara et al. [7]

**Fig. 2:**
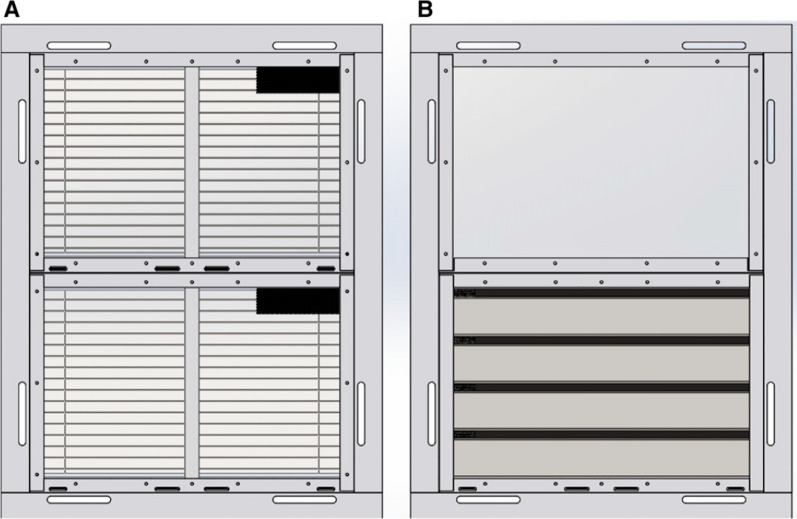
Prototype windows (internal view). Where **A** is the blinds window and **B** is a window combining a translucent panel at the top of the concertinaed panel at the bottom. Source: Jawara et al. [7]

In 2017, the protective efficacy of four prototype screened doors and two window designs against mosquito entry (Figs. 1 and 2) was measured, as well as their short-term durability and acceptability [7]. These doors and windows had several important features. Firstly, they were constructed entirely from metal, apart from two of the door prototypes and one window which had a single panel of translucent polycarbonate to allow light to enter the building when doors and windows were closed. Secondly, the doors were self-closing and the windows fixed in the closed position. Thirdly, the doors had lever handles that could be locked internally by moving a small lever on the lock.

In this original study, two doors and two windows were installed in 24 randomly selected houses in a Gambian village, reducing mosquito house entry by 59–77%. Most of the door openings occurred at night (79%) from dusk to midnight, when malaria vectors begin entering houses. The indoor climate of houses with screened doors was similar to control houses, although in all houses, based on human comfort indices, it was overall too hot before midnight and too cold after midnight. Ten weeks after installation, the doors and windows were in good condition, although 38% of doors did not fully self-close and latch (snap shut). The new doors and windows were popular with local residents. At the end of the rainy season in 2017, additional doors and windows were installed in the six control houses and in the Alkalloh’s (village leader) house (n = 31).

After 4 years use in the villages, the team returned to the same village and recorded the durability and use of the prototype doors and windows and canvassed the opinions of the house owners on the intervention, and how they might be improved. No entomological data were collected since houses previously used as controls subsequently had screening installed. Although this is a small study, follow up surveys are rarely done with screening interventions. This report will be of interest to those working on building interventions to improve health. These findings are relevant to those interested in house screening for malaria control in The Gambia and other parts of sub-Saharan Africa.

## Methods

### Study design

A cross-sectional survey was conducted 4 years after the installation of novel prototype screened doors and windows in a Gambian village. A questionnaire survey was employed to record the durability and functionality of the doors and windows and to assess the responses of house owners to this intervention.

### Study area

The survey was carried out in Wellingara village (N 13° 33.3659′, W 14° 55.4619′) on the south bank of the River Gambia in Lower Fulladu West, Central River Region, from 5 to 8th August 2021, at the beginning of the rainy season, a period of heavy rain. Research staff revisited all 31 screened houses: 24 screened at the start of the original study in August 2017, plus the six control houses and Alkhalloh’s (village leader) house screened at the end of the trial in November 2017.

### Study procedures

**Fig. 3:**
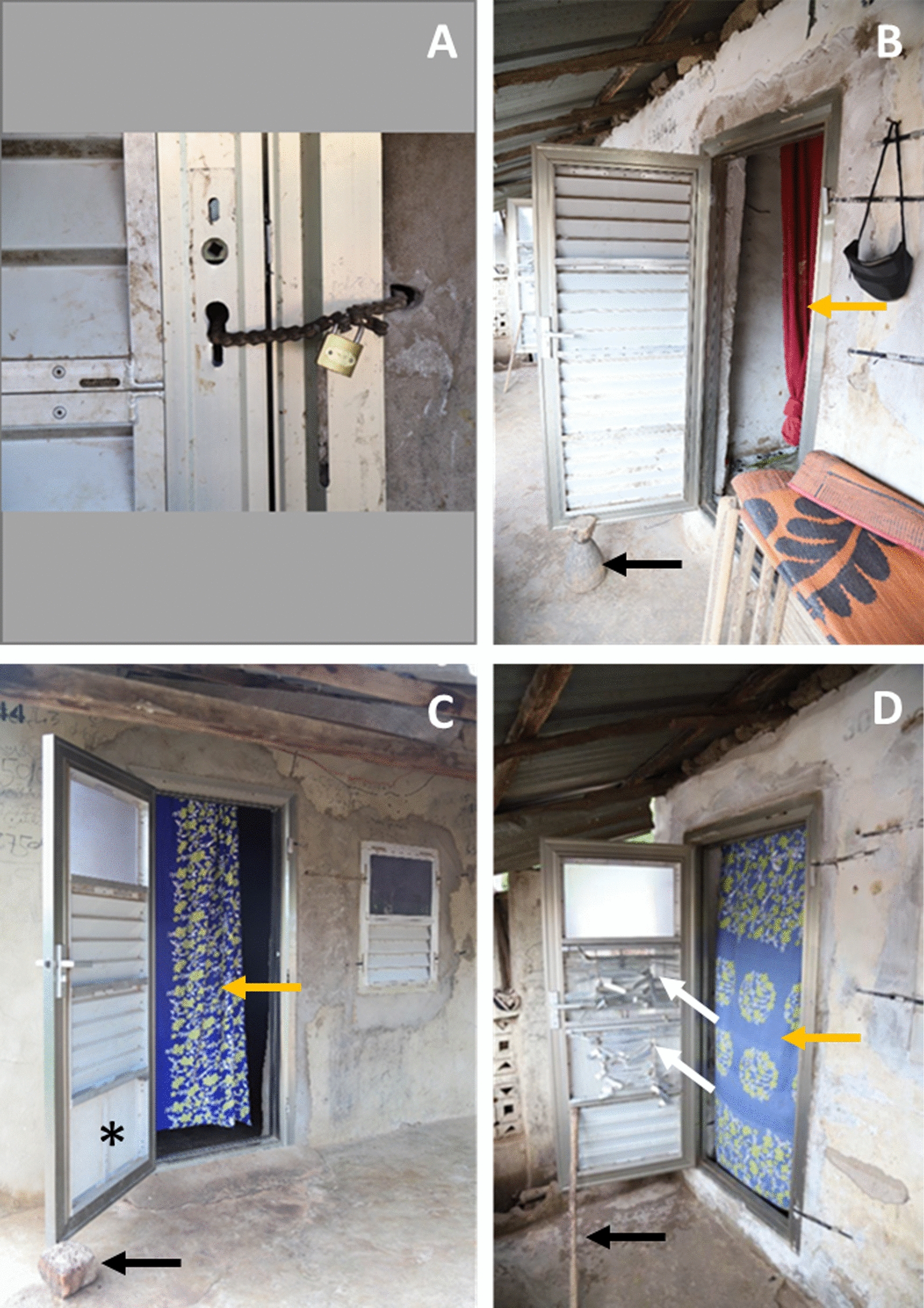
Examples of door usage showing modifications and damage to blinds. **A** The door handle has been lost and the home owners have made a hole in the wall to use a bicycle chain and padlock to secure the door; **B** door propped open to prevent automatic shutting (black arrow); **C** loss of blinds in the lower panel (asterix) and the door kept ajar during the day with a brick (black arrow); **D** damaged blinds in the two middle door panels (white arrows) with the door get open with a stick (black arrow). Note brightly coloured curtains (orange arrows **B**–**D**)

Durability and functionality of the doors and windows was evaluated using a survey form similar to the one used previously (Additional file 1). The structural condition and cleanliness of the prototype doors, windows, blinds and their frames were assessed. Whether the doors shut automatically, smoothly and completely was also recorded, and the condition of the locks was noted. In addition, the room owner (or on a few occasions their representative) was asked in Mandinka, the local language, their opinion of the doors and windows, namely ‘*What do you like best about the new doors and windows*?’ and ‘*How do you think they could be improved*?’ Finally, having noted in 2017 that many households chose to hang curtains inside their doors and windows (Fig. 3 panels B, C and D), curtain usage was recorded and the reason for putting up curtains was asked (Additional file 2). All responses were immediately translated into English and recorded onto the survey form.

### Data analyses

This was a descriptive survey and no statistical comparisons were made between different prototypes because of the small sample sizes. Findings were summarized as percentages.

## Results

### Condition and functionality of screened doors and windows

Thirty-one house units, each with 2 doors and 2 windows were surveyed (Table 1). One house was locked (so that only the front door and window were accessible) and one was vacated and being used as a rice store, but still accessible. There was no damage to the doors and windows or their frames. Very minor, mostly hair-line cracks were commonly seen in the rendering around the doors and windows. In two houses a hole had been made deliberately in the wall on the lock side to pass a chain which could be padlocked, through the wall and the missing door lock (Fig. 3, panel A). Gaps between the wall and the frames were recorded in just 2/61 (3%) doors and 1/61 (2%) windows. Only 50/92 (54%) of doors and windows (back windows not checked) were reported as clean; however, this was mostly light dust/mud and only one unit had a very dirty front door and window (the rice store).

**Table 1:** Condition and functionality of doors and windows

Characteristic	Position of door or window		
	Front	Back	Front and back
Doors			
Number of doors examined	31	30	61
Door propped open at time of visit	21/31 (68%)	nd	
Door clean	14/31 (45%)	22/30 (73%)	36/61 (59%)
Mortar around the frames undamaged^a^	26/31 (84%)	30/30 (100%)	56/61 (92%)
Gaps	1/31 (3%)	1/30 (3%)	2/61 (3%)
Door undamaged	31/31 (100%)	30/30 (100%)	61/61 (100%)
Door closed smoothly	29/31 (94%)	21/30 (70%)	50/61 (82%)
Door fully shut automatically	17/31 (55%) (+ 10 didn’t snap shut)	8/30 (27%) (+ 8 didn’t snap shut)	25/61 (41%) (+ 18 didn’t snap shut)
Original door lock present and working	14/31 (45%)	10/30 (33%)	24/61 (39%)
Door lock replaced with padlock^b^	3/31 (10%)	1/30^c^ (3%)	4/61 (7%)
Evidence of a trace of water inside	0/61 (0%)	3/30 (10%)	3/61 (5%)
Door with original blinds in working order	1/55 (2%)	3/54 (6%)	4/109 (4%)
Curtains present	27/31 (87%)	23/30 (77%)	50/61 (82%)
Windows			
Number of windows examined	31	30	61
Window clean	14/31 (45%)	nd	
Mortar around the window frames undamaged^a^	31/31 (100%)	30/30 (100%)	61/61 (100%)
Gaps present around frames	1/31 (3%)	0/30 (0%)	1/61 (2%)
Window undamaged	31/31 (100%)	30/30 (100%)	61/61 (100%)
Evidence of water inside	0/31 (0%)	nd	
Window with original blinds in working order	6/29 (21%)	4/27 (15%)	10/56 (18%)
Curtains present	10/31 (32%)	16/30 (53%)	26/61 (43%)

nd: not done

^a^Very minor, hairline superficial cracks in the rendering (most likely there from the outset) were discounted

^b^In two houses a hole had been made deliberately on the lock side so a chain through the wall and a missing door lock could be padlocked (Fig. 3A)

^c^Locked with a small stick

Most front doors (21/31, 68%) were found propped open (Fig. 3, panels B and C). Most front doors (29/31, 94%) and back doors (21/30, 70%) closed smoothly, but only 17/31 (55%) of front doors and 8/30 (27%) of back doors could fully shut automatically. Several doors failed to snap shut (front doors 10/31, 32%; back doors 8/30, 27%). The main reason for doors not closing smoothly was due to sticking on the bottom right-hand side because the door was hanging away from the hinges on the opposite side (7/11, 64%). Door locks proved less sturdy than the doors themselves, with only 24/61 (39%) present and working. The blinds proved especially flimsy (Fig. 3, panels C and D), with only 4/109 (4%) of door blinds and 10/56 (18%) of window blinds present and in working order. There was no sign of water entering through front doors, whereas three (3/30, 10%) of the back doors had trace amounts of water inside. None of the front windows had allowed in water (back windows were not checked).

Householders hung curtains inside most doors (50/61, 82%) (Fig. 3, panels B, C and D) and in 26/61 (43%) of windows. Most of those with a front door curtain 23/27 (85%), put their curtains down at night.

### Acceptability

Doors and windows were well liked (Table 2), with 19/31 (61%) of respondents professing to be happy with them. The main reasons cited were that they kept mosquitoes out 14/31 (45% of respondents) and provided security 12/31 (39%). There were complaints about the poor quality of the locks and door handles (15 responses) (the lock and handle were combined in the same cassette). Respondents were also dissatisfied with the fragile blinds (8 responses). Eight people wanted windows that could be opened and five said that the houses were too hot. One specifically mentioned lack of ventilation and another expressed a preference for the ridged door (i.e. the concertinaed door Fig. 1A). Three householders claimed that the back doors let water in.

**Table 2:** Acceptability of doors and windows and reasons for hanging curtains

Reasons for liking screened doors and windows	No.	Improvements needed/dislikes doors and windows	No.	Reasons for curtains	No.
Liked in general	19	Poor-quality locks	9	Privacy, especially when the door is open	26
Keeping mosquitoes out	14	Windows that open	8	To keep light out	7
Security	12	Fragile blinds	8	For warmth (when they are down)	3
Keeping flies/insects out	7	Poor-quality door handles	6	For ventilation (when they are up)	3
Look beautiful	3	Made the room too hot	5	To protect against thunder and lightning	3
Ventilation	2	Let water in	3		
Strength	1	Prefer ridged door [like Fig. 1A]	1		
Less malaria	1	Lack of ventilation	1		
Prevents dust	1				
Prevents snakes and scorpions	1				

The most common reason given for the use of curtains was to provide privacy 26/28 (93% of those with curtains), especially while the door was open (Table 1). Seven said the curtains were used to keep light out.

Interviews with room owners illustrated why people liked the doors and windows saying:

*"I like them because windows are secure and beautiful and people appreciate them especially if they are cleaned. They are secured and protected."*

*"Mosquitoes doesn’t enter and it’s very safe."*

*"I am happy about the doors and windows, when they are closed nothing can go in."*

*"Doors and windows protect us from flies, mosquitoes and other insects. If it is locked nothing to enter. We are grateful to Allah because we have the doors."*

Respondents also suggested improvements to the design of the doors and windows including:

*"Handles and locks are bad, I would like replacements."*

*"In hot weather, window should open."*

*"The blinds are not good. Rain enters through the back door, through the screen because there are no blinds."*

*"The only problem is the windows don’t open."*

*"The only thing. The blinds should be stronger."*

There were a variety of explanations given for the use of curtains behind the doors and windows, including:

*"The reason for a curtain is to provide privacy, when the door is open people will not see inside."*

*"I don’t want anybody to see inside, while outside."*

*"To prevent light penetrating inside and also my privacy is important, while the door is open."*

*"To prevent thunder and lightning and also for privacy."*

*"I place my curtain for privacy and when it is hot I do hang it [up], when it is cold I bring it down to get some heat."*

*"To cover-up light flashing inside the house during thundering and when the door is open people will not see inside."*

*"To prevent people seeing inside the house whenever it [the door] is open and to avoid light penetration."*

## Discussion

This study is the longest follow up of the durability and functionality of house modifications designed to reduce the entry of malaria mosquitoes. The doors and windows themselves proved very robust, with no damage recorded to either them or their frames. Most looked new and were clean suggesting that the owners took good care of them. Although minor cracks were noted in the mortar around doors and windows, these were most likely present from the time they were first installed (“Hairlike cracks were apparent in some concreting around the door……” [7]). Hairline cracks are a common occurrence when cement and mud are married, as in most rural construction work in The Gambia. They appear as the cement dries in the heat.

There were several features of the doors that should be improved or removed. Firstly, only 39% of the door lock handles were present and in working order. The lock mechanisms commonly failed, resulting in homeowners removing the handles so that the doors could be secured with a chain and padlock. Stronger lock handles are needed, or they could be substituted with simpler solutions, such as using bolts internally and hasp and staple locks with padlocks externally. Although the use of padlocks externally is a common practice in The Gambia and other countries in sub-Saharan Africa, it is potentially dangerous since individuals could be locked indoors and unable to exit in a fire. Secondly, the blinds were not fit for purpose, with only 4% of door blinds and 18% of window blinds still present and working. Of the four prototype doors it is the concertinaed door (Fig. 1A), which has no blinds and could be mass-produced from a single sheet of metal, which would be the most robust prototype recommended for village use. Thirdly, although 82% of the self-closing doors closed smoothly only 41% of doors fully shut automatically. Of the few doors which stuck (11 in total), most 7/11 (64%) were catching on the bottom right-hand side. Such damage may have been caused by children swinging on the doors and pulling them away from their hinges on the opposite side or even bending the door blade itself. Alternatively, it could be due to slight shifting of the doors after they were first installed, before the cement set. Room owners were asked not to open doors for at least 48 h following installation [7], but this may not have always been strictly adhered to. The doors are designed to close automatically by using spring-loaded hinges. The idea behind this is to facilitate closing the doors at night to reduce the number of mosquitoes entering the house. Yet, in keeping with observations in the original study, most front doors (68%) were found to be propped open with sticks or a heavy object (brick or stool) to allow easy access into the home and to promote cross ventilation. Jawara et al. noted “that generally the self-closing prototype doors are open for much shorter periods than control doors, although after 06.00 h, with the prototype doors, some doors are propped open to allow housework to take place unimpeded.” In common with many interventions, health messaging is also required. In this case, house screening needs to be accompanied with strong messaging on keeping doors and windows closed at night.

The novel screened doors and windows were well liked in general and particularly for their ability to keep mosquitoes and other insects out and offer security. This was broadly in agreement with findings from the initial intervention where across all Focus Group Discussions (FGDs), participants mentioned that the doors provided privacy, kept out mosquitoes and were attractive to look at [7]. Indeed, the general view amongst the community was that the doors and windows were beautiful and were a major improvement to their homes. The most frequent complaints concerned the poor quality of the lock handles and the blinds.

There were requests for windows which could be opened, five respondents said that their houses were too hot and one mentioned lack of ventilation. Some occupants told MCT that the houses became too hot when the metal doors had been closed all day, while they were away working in the fields. When they returned and opened the door, they were met by a wave of heat. The original study however, showed that there was no difference in indoor temperatures between control and intervention units. Study houses were almost entirely outside the human comfort index for most of the night, being too hot before midnight and too cold after midnight [7]. Also, in the original study, most FGD respondents liked the windows because they allowed ventilation and looked attractive. Respondents noticed that *“fresh air enters the house due to the holes”* and it is *“beautiful just like the doors.”* Nevertheless in some FGDs, people were concerned about the windows not opening: *“The major problem with the windows is their lack of opening but had it been they open and close it would be the best.”*

Three respondents in our follow up survey complained of back doors allowing water ingress. Since all the houses had front verandas, front doors were protected from the rain by the overhanging roof. Only two of the three who complained about water inside the house had any sign of water being present (trace inside back doors), also they were responding during a particularly heavy downpour so were conceivably influenced by the heavy rain, strong wind and flooding outdoors when interviewed.

Most people hung curtains in their doors (82%) and in many of their windows (43%). The most common explanation for the curtains was to provide privacy, especially when the door was open. Seven said to keep light out, especially when there was lightning during the heavy storms that occur chiefly during the early rainy season at the time of interview. The fear of lightning probably stems from the danger of being physically injured or the house being burnt down as can happen with thatched-roofed houses. Although not stated, it appeared to the investigators that curtains were used in part to decorate the house since they were brightly coloured, individualized and could be easily seen. It is also likely that people use curtains for privacy, rather than closing the door which may be considered rude, and to allow small children to come in and out of the house more easily. Having the curtains down inside the doors will reduce the amount of dust that enters the house during the dry season when strong Harmattan winds bring dust down from the Sahara but will also impede ventilation in the house. A well-ventilated house is important since it will help keep the house cool at night and increase the likelihood of people using a mosquito net [8, 9], it is also likely to reduce mosquito house entry [9] and reduce the incidence of respiratory disease [10].

This study has several limitations. Firstly, the sample size is small with new doors and windows installed in just 31 house units. Nonetheless the findings were replicated across units with different prototype doors and windows. Secondly, the acceptability survey is susceptible to social desirability bias since some house owners may have either given answers they believed the researchers expected or just wished to be polite. Having been given brand new doors and windows, they may have been unwilling to offend by voicing criticisms of the intervention.

## Conclusions

The prototype doors and windows proved highly durable, even after 4 years in use. This is one advantage over current vector control tools, such as the use of indoor residual spraying which is usually done annually [11] or insecticide-treated bed nets, where relatively few nets remain in good condition for 3 years in the field [12]. That said, screening, unlike the use of insecticides, will not kill mosquitoes but can still provide protection to those sleeping indoors and for this reason is an intervention endorsed by the WHO [1]. Improvements to the design include: making doors from single-sheets of pressed metal and using simpler and more robust door locks. Health promotion should be an important component of house screening programmes and include advice on closing the doors at night and not to block ventilation by covering the screened doors with curtains. The development of new types of screened doors and windows, if used correctly, could contribute to sustainable malaria control in sub-Saharan Africa.

## Supplementary Information

**Additional file 1.** Screened doors survey form 2021 v1.1.

**Additional file 2.** Curtain survey.

## Data Availability

The datasets used for the study are available from the corresponding author on reasonable request.

## Abbreviation

FGD: Focus group discussions

## References

[1] WHO (2021). Guidelines for malaria.

[2] Ogoma SB, Kannady K, Sikulu M, Chaki PP, Govella NJ, Mukabana WR (2009). Window screening, ceilings and closed eaves as sustainable ways to control malaria in Dar es Salaam, Tanzania. Malar J.

[3] Killeen GF, Govella NJ, Mlacha YP, Chaki PP (2019). Suppression of malaria vector densities and human infection prevalence associated with scale-up of mosquito-proofed housing in Dar es Salaam, Tanzania: re-analysis of an observational series of parasitological and entomological surveys. Lancet Planet Health.

[4] Kaindoa EW, Finda M, Kiplagat J, Mkandawile G, Nyoni A, Coetzee M (2018). Housing gaps, mosquitoes and public viewpoints: a mixed methods assessment of relationships between house characteristics, malaria vector biting risk and community perspectives in rural Tanzania. Malar J.

[5] Kirby MJ, Bah P, Jones CO, Kelly AH, Jasseh M, Lindsay SW (2010). Social acceptability and durability of two different house screening interventions against exposure to malaria vectors, *Plasmodium falciparum* infection, and anemia in children in the Gambia, West Africa. Am J Trop Med Hyg.

[6] Getawen SK, Ashine T, Massebo F, Woldeyes D, Lindtjorn B (2018). Exploring the impact of house screening intervention on entomological indices and incidence of malaria in Arba Minch town, southwest Ethiopia: a randomized control trial. Acta Trop.

[7] Jawara M, Jatta E, Bell D, Burkot TR, Bradley J, Hunt V (2018). New prototype screened doors and windows for excluding mosquitoes from houses: a pilot study in rural Gambia. Am J Trop Med Hyg.

[8] Pulford J, Hetzel MW, Bryant M, Siba PM, Mueller I (2011). Reported reasons for not using a mosquito net when one is available: a review of the published literature. Malar J.

[9] Jatta E, Carrasco-Tenezaca M, Jawara M, Bradley J, Ceesay J, D’Alessandro U (2021). Impact of increased ventilation on indoor temperature and malaria mosquito density: an experimental study in The Gambia. J R Soc Interface.

[10] WHO (2018). Housing and health guidelines.

[11] Najera JA, Zaim M (2001). Malaria vector control. Insecticides for indoor residual spraying.

[12] Lindsay SW, Thomas MB, Kleinschmidt I (2021). Threats to the efficacy of insecticide-treated bednets for malaria control: thinking beyond insecticide resistance. Lancet Glob Health.

