# Supplementary material for: The durability, functionality and acceptability of novel screened doors and windows after 4 years of use in a Gambian village: a cross-sectional survey
Source: Malar J. 2022 Feb 23;21:64. doi: 10.1186/s12936-022-04087-9 (PMC8867667; doi:10.1186/s12936-022-04087-9)
Supplement: Supplementary file 1 — Additional file 1. Screened doors survey form 2021 v1.1. [file 12936_2022_4087_MOESM1_ESM.docx]

**SCC 1390 TOWARDS THE END GAME: OPERATIONAL RESEARCH ON IMPROVING RURAL HOUSING IN SUB-SAHARAN AFRICA AS A STRATEGY TO SUPPORT MALARIA ELIMINATION**

# Door and Window Durability Survey

**WELLINGARA PILOT DOOR STUDY (2021)**

|  | **GENERAL INFORMATION (TO BE COMPLETED IN FIELD)** |
| --- | --- |
| 1 | Date of Collection: \|___\|___\|/\|___\|___\|/\|___\|___\|___\|___\| (dd/mm/yyyy) |
| 2 | Week number: \|___\|___\| (e.g. 01) |
| 3 | Compound Number: \|___\|___\|___\| (e.g. 012) |
| 4 | Name of Compound Head : |
| 5 | House Number: \|___\|___\| (As in randomization list) |
| 6 | Unit Number \|___\|___\| (Number painted near the door e.g. 12) |
| 7 | Name of House Dweller/Owner : |
| 8 | Door type (D1, D2, D3 or D4): \|___\|___\| |
| 9 | Window type (W1, W2): \|___\|___\| |
| 10 | Number of people sleeping in the Unit \|___\| |
| 11 | Number of adults over 15 years \|___\| |
| 12 | Number of children under 15 years \|___\| |
| 13 | Number of beds: \|___\| |
| 14 | Number of bed nets in use: \|___\| |
| 15 | Are these all LLIN? : Y/N \|___\| |
| 16 | Record recent decoration or improvement indoors or outdoors (*write none if none*) |

|  | **FRONT DOOR** |
| --- | --- |
| 17 | Record damage to house around door e.g. cracks etc |
| 18 | Did this happen since the door was installed? Y/N \|___\| |
| 19 | Record any damage to the door frame Y/N \|___\| |
| 20 | Did you find the door propped open? |
| 21 | Is their evidence of water inside the door? Y/N \|___\| |
| 22 | Is the door clean? Y/N \|___\| |
| 23 | Is their damage to the door structure? |
| 24 | If yes, describe |
| 25 | Does the door shut automatically? Y/N \|___\| |
| 26 | Does the door open and shut smoothly? Y/N \|___\| |
| 27 | If not, why not? |
| 28 | Is the door lockable? Y/N \|___\| |
| 29 | Is the door locked daily? Y/N \|___\| |
| 30 | Does the owner still have two keys? Y/N \|___\| |
| 31 | Record problems with locks |
| 32 | When the door is shut are there gaps around the door? Y/N \|___\| |
| 33 | If yes, record size: \|___\| . \|___\| (cm). |
| 34 | Position: top / bottom / hinge side / lock side. (*Please circle*) |
| 35 | If yes, record size: \|___\| . \|___\| (cm). |
| 36 | Position: top / bottom / hinge side / lock side. (*Please circle*) |
| 37 | Should the door have blinds? Y/N \|___\| , *(if No skip to next section)* |
| 38 | How many blinds should there be? \|___\| |
| 39 | How many blinds are present? \|___\| |
| 40 | How many blinds are functioning correctly? \|___\| |
| 41 | Describe type of damage to door |

|  | **BACK DOOR** |
| --- | --- |
| 42 | Record damage to house around door |
| 43 | Did this happen since the door was installed? Y/N \|___\| |
| 44 | Record any damage to the door frame: ………………………………………………………  ……………………………………………………………………………………………………… |
| 45 | Is their evidence of water inside the door? Y/N \|___\| |
| 46 | Is the door clean? Y/N \|___\| |
| 47 | Does the door shut automatically? Y/N \|___\| |
| 48 | Does the door open and shut smoothly? Y/N \|___\| |
| 49 | If not, why not? |
| 50 | Is the door lockable? Y/N \|___\| |
| 51 | Is the door locked daily? Y/N \|___\| |
| 52 | Does the owner still have the key? Y/N \|___\| |
| 53 | When the door is shut are there gaps around the door? Y/N \|___\| |
| 54 | If yes, record size: \|___\| . \|___\| (cm). |
| 55 | Position: top / bottom / hinge side / lock side. (*Please circle*) |
| 56 | If yes, record size: \|___\| . \|___\| (cm). |
| 57 | Position: top / bottom / hinge side / lock side. (*Please circle*) |
| 58 | Should the door have blinds? Y/N \|___\| , *(if No skip to next section)* |
| 59 | How many blinds should there be? \|___\| |
| 60 | How many blinds are present? \|___\| |
| 61 | How many blinds are functioning correctly? \|___\| |
| 62 | Describe type of damage to door |

|  | **FRONT WINDOW** |
| --- | --- |
| 63 | Record damage to house around window |
| 64 | Did this happen since the window was installed? Y/N \|___\| |
| 65 | Record any damage to the window frame: ………………………………………………………  ……………………………………………………………………………………………………… |
| 66 | Is their evidence of water inside the window? Y/N \|___\| |
| 67 | Is the window clean? Y/N \|___\| |
| 68 | Are there gaps around the window? Y/N \|___\| |
| 69 | If yes, record size: \|___\| . \|___\| (cm) |
| 70 | Position: top / bottom / hinge side / lock side. (*Please circle*) |
| 71 | If yes, record size: \|___\| . \|___\| (cm). |
| 72 | Position: top / bottom / hinge side / lock side. (*Please circle*) |
| 73 | Does the window have blinds? Y/N \|___\| , *(if No skip to next section)* |
| 74 | How many blinds should there be? \|___\| |
| 75 | How many blinds are present? \|___\| |
| 76 | How many blinds are functioning correctly? \|___\| |

|  | **BACK WINDOW** |
| --- | --- |
| 77 | Record damage to house around door |
| 78 | Did this happen since the door was installed? Y/N \|___\| |
| 79 | Record any damage to the window frame: ………………………………………………………  ……………………………………………………………………………………………………… |
| 80 | Are there gaps around the window? Y/N \|___\| |
| 81 | If yes, record size: \|___\| . \|___\| (cm) |
| 82 | Position: top / bottom / hinge side / lock side. (*Please circle*) |
| 83 | If yes, record size: \|___\| . \|___\| (cm). |
| 84 | Position: top / bottom / hinge side / lock side. (*Please circle*) |
| 85 | Does the window have blinds? Y/N \|___\| , *(if No skip to next section)* |
| 86 | How many blinds should there be? \|___\| |
| 87 | How many blinds are present? \|___\| |
| 88 | How many blinds are functioning correctly? \|___\| |
| 89  90 | What do you like best about the new doors and windows?  How do you think they could be improved? |

Field Assistant signature and initials: _____________________ Initials |____|____|
